# Amyloid burden, brain metabolism, and gray matter volume in SuperAgers

**DOI:** 10.1055/s-0045-1814400

**Published:** 2026-02-04

**Authors:** Adalberto Studart-Neto, Artur Martins Coutinho, Camila de Godoi Carneiro, Natália Cristina Moraes, Jacy Bezerra Parmera, Milena Sales Pitombeira, Daniele de Paula Faria, Raphael Ribeiro Spera, Mateus Rozalem Aranha, Carla Rachel Ono, Omar Jaluul, Mônica Sanches Yassuda, Claudia da Costa Leite, Sonia Maria Dozzi Brucki, Carlos Alberto Buchpiguel, Ricardo Nitrini

**Affiliations:** 1 Universidade de São Paulo, Faculdade de Medicina, Hospital das Clínicas, Departamento de Neurologia, São Paulo SP, Brazil.; 2 Universidade de São Paulo, Faculdade de Medicina, Hospital das Clínicas, Departamento de Radiologia e Oncologia, Divisão e Laboratório de Medicina Nuclear (LIM 43), São Paulo SP, Brazil.; 3 Universidade de São Paulo, Faculdade de Medicina, Hospital das Clínicas, Departamento de Radiologia e Oncologia, Laboratório de Investigação Médica em Radiologia (LIM 44), São Paulo SP, Brazil.; 4 Universidade de São Paulo, Faculdade de Medicina, Hospital das Clínicas, Serviço de Geriatria, São Paulo SP, Brazil.; 5 Universidade de São Paulo, Escola de Artes, Ciências e Humanidades, São Paulo SP, Brazil.

**Keywords:** Memory, Amyloid, Positron-Emission Tomography, Magnetic Resonance Imaging, Aging

## Abstract

**Background:**

*SuperAgers* (SA) are adults aged ≥ 80 years with memory equivalent to individuals 20 to 30 years younger. Few studies have evaluated multimodal neuroimaging approach in the same SA cohort.

**Objective:**

To investigate neurobiological mechanisms underlying exceptional cognitive aging by evaluating cortical amyloid deposition, regional cerebral glucose metabolism (rBGM), and gray matter volume (GMV), and their associations with neuropsychological performance and subjective cognitive decline (SCD).

**Methods:**

The participants were classified as SA (n = 11), age-matched healthy controls (HC80; n = 23), and healthy controls aged 60 to 69 years (HC60; n = 23). Positron-emission tomography (PET) using ^11^ C-PIB and ^18^ F-FDG were analyzed using semiquantitative three-dimensional stereotactic surface projection (3D-SSP), with group comparisons using Statistical Parametric Mapping 8 (SPM8).

**Results:**

The median ages were 81 years (interquartile range [IQR] = 5.0) for SA, 83 years (IQR = 5.0) for HC80, and 66 years (IQR = 3.0) for HC60. All groups had a median of 16 years of schooling (IQR for SA = 7, for HC80 and HC60 = 5). There were 4 PIB-PET positive individuals (36.4%) in the SA group, which is similar to the HC80 group (40.9%). Also, 6 SA had SCD, with 3 being PIB-positive. In SA, composite SUVr predicted RAVLT delayed recall (β = −0.666, *p* = 0.011, adjusted R ^2^ = 0.748), controlling for age, sex, and schooling. Compared to HC80, the SA group showed increased metabolism in the anterior cingulate gyrus and caudate, as well as increased GMV in the putamen.

**Conclusion:**

The SA group exhibited similar amyloid burden to HC80, yet amyloid deposition specifically impaired their memory. Increased rBGM and GMV in the salience network and striatum suggest these regions support successful cognitive aging.

## INTRODUCTION

Most individuals experience gradual cognitive decline associated with aging, and episodic memory is one of the most affected domains. 1 There is a growing interest in studying older adults whose cognitive abilities remain equivalent to middle-aged adults and understanding the mechanisms of resilience to aging and neurodegenerative pathologies. 2 3 4 5

The term *SuperAgers* (SA) was proposed to define older adults with 80 years and older who have a memory performance equivalent to individuals between 20 and 30 years younger, according to the criteria established by the Northwestern SuperAging research program. 6 7 Neuroimaging studies have been looking for biological markers that differentiate SA from age-matched older adults. Cortical morphometry methods on magnetic resonance imaging (MRI) showed that SA have less age-related cortical atrophy, greater cortical thickness in the anterior cingulate gyrus, and larger hippocampal volume. 4 6 7 8

Additionally, in positron-emission tomography (PET) studies, SA showed no difference in amyloid burden and lower tau deposition compared to age-matched older adults. 8 9 10 However, this condition is relatively rare, and existing studies have typically included small samples from high-income countries, limiting the generalizability of findings. Notably, little is known about whether the neurobiological markers observed in SA—such as less cortical atrophy, preserved glucose metabolism, and reduced tau deposition—are consistent across diverse sociocultural and environmental contexts, particularly in low- and middle-income countries. Furthermore, few studies have simultaneously examined multiple neuroimaging biomarkers in a single SA cohort.

This study aimed to advance understanding of the neurobiological mechanisms associated with exceptional cognitive aging through a multimodal neuroimaging approach, evaluating cortical amyloid deposition, regional cerebral glucose metabolism (rBGM), and gray matter volume (GMV), as well as examining the relationship of these biomarkers with both objective neuropsychological performance and subjective cognitive complaints in SA from a middle-income country. This integrated analysis provides a comprehensive and contextualized neurobiological characterization of this population, which is still underrepresented in the literature.

## METHODS

### Participants

Participants were classified into three groups: SA (age ≥ 80 years), age-matched healthy controls (HC80), and healthy controls aged 60 to 69 years (HC60). All participants were required to meet the following inclusion criteria: schooling ≥ 4 years; Mini-Mental State Examination (MMSE) results within normal range average for their schooling; 11 12 and functional activity questionnaire score (FAQ) ≤ 4. 13

We defined SA according to the established criteria, 6 7 which indicate participants should perform at or above the normative values determined for individuals between 50 and 65 years of age on the Brazilian version of the Rey Auditory Verbal Learning test (RAVLT) delayed recall (RAVLT-DR) score (≥ 10 words), 14 and perform within or above 1 standard deviation (SD) of the mean score for their age and schooling on nonmemory domains tests, including executive function, attention, language, and visuospatial abilities, as detailed in the next section. Participants aged ≥ 80 years with a RAVLT-DR score of less than 10 words were included in the HC80 group. They could present any RAVLT-DR score considered normal for sex- and age-normalized data.

The exclusion criteria were: diagnosis of dementia or mild cognitive impairment (MCI); 15 16 diagnosis of a major psychiatric disorder; 17 history of alcohol or psychoactive drug abuse; current or previous diagnosis of neurological diseases; and visual or auditory limitations that could impair performance in cognitive tests.

Initially, 140 participants were recruited in the city of São Paulo (Brazil): the outpatient Clinic for “Elderly without Systemic and Symptomatic Senility” at the Geriatrics Service of Hospital das Clínicas da Faculdade de Medicina da Universidade de São Paulo, Brazil (HCFMUSP); The Open University Program for Senior Citizens at Escola de Artes, Ciências e Humanidades da Universidade de São Paulo; and a Development Center for the Promotion of Healthy Aging.

**Figure 1:**
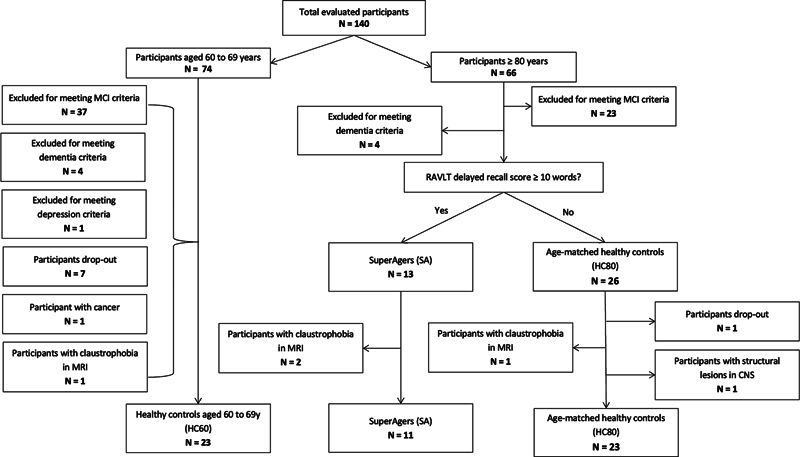
Abbreviations: MCI, mild cognitive impairment; MRI, magnetic resonance imaging; PET, positron-emission tomography; RAVLT, Rey Auditory Verbal Learning Test. Flowchart of the initial sample and the final sample of participants who underwent FDG-PET-MRI and PIB-PET-MRI imaging.

We also recruited community elders through social media and newspapers. In total, 57 participants were included, and the sample classification across the three groups is summarized in Figure 1 . There were 11 SA participants in the final sample.

### Neuropsychological assessment

The first assessment consisted of sociodemographic data, MMSE, the Montreal Cognitive Assessment (MoCA), the Brief Cognitive Screening Battery (BCSB), which includes the Figure Memory Test (FMT); 18 19 15-question version of the Geriatric Depression Scale, the Geriatric Anxiety Inventory, and FAQ. The question “do you feel like your memory is becoming worse?” was carried out for all participants; those who answered “yes” were considered to have a subjective cognitive decline (SCD). 20

After that, the participants who met the inclusion criteria completed neuropsychological tests standardized for use in the Brazilian population. The tests performed were: Forward and Backward Digit Span Test, Trail Making A (TMA) and B (TMB), Phonological Verbal Fluency (FAS), Rey-Osterrieth Complex Figure (copy and delayed recall), Logical Memory of the Wechsler Memory Scale, RAVLT and Boston Naming Test (BNT). The estimated Intelligence Quotient (IQ) was measured with Wechsler Adult Intelligence Scale, Third Edition (WAIS-III).

We used the operational criterion adopted by Petersen et al. for defining MCI (at least one neuropsychological test with a result equal to or less than −1.5 SDs from average normative values adjusted by age and schooling). 21 Using this criterion, 60 participants were classified as having MCI and excluded.

### Imaging data acquisition

The Pittsburgh compound B (PIB) and fluorodesoxiglicose (FDG) PET images were acquired on separate days, each performed simultaneously with MRI on a 3.0-Tesla SIGNA (GE HealthCare) PET/MRI hybrid scanner. The average acquisition interval between the FDG and PIB-PET images was 18.5 ± 17.9 days.

The MRI protocol included volumetric T1- and T2-weighted, fluid-attenuated inversion recovery (FLAIR), and susceptibility-weighted imaging (SWI) sequences. No paramagnetic intravenous contrast material was used. Images were visually inspected by a neuroradiologist to detect structural brain lesions, artifacts that could impair imaging processing, and the assessment of white matter hyperintensities (WMH), according to the Fazekas scale. 22

There were ^11^ C-PIB and ^18^ F-FDG produced in the on-site cyclotron; details of the production process, including appropriate quality control measures, were previously described. 23 The FDG was injected in bolus (208.0 ± 17.9 MBq), and participants remained at rest, without audiovisual stimuli and eyes open, for 30 minutes before imaging acquisition.

The PIB-PET images were acquired during 70 minutes with a dynamic protocol started immediately after intravenous administration of PIB (378.2 ± 37.5 MBq). The late phase images were analyzed (acquisition time: 50–70 min postinjection), reconstructed and condensed in time for the creation of single static images.

### PIB-PET individual analysis and visual classification

The visual and semi-quantitative approaches used for individual classification were previously published. 24 Two nuclear medicine physicians proceeded with the PIB-PET images visual assessment, blinded to each other's interpretation and group classification. In cases of discordance, we conducted a consensus reading with the aid of the three-dimensional dimensional stereotactic surface projection program (3D-SSP) semi-quantitative method.

Each PIB-PET scan was classified as “amyloid positive” if there was a loss of gray and white matter contrast, with increased uptake in cortical gray matter in at least two of the following six areas: frontal, temporal, lateral parietal, precuneus, anterior cingulate, and posterior cingulate. Furthermore, the image was classified as positive if only a single large cortical area had a strong tracer uptake. Conversely, the image was rated as “amyloid negative” when there was a clear contrast between gray and white matter, with strong uptake in the white matter and no significant uptake in the cortex.

Semiquantitative analysis was performed using the 3D-SSP method with the Cortex ID Suite software (GE Healthcare). The composite standardized uptake values ratio (SUVr) was calculated using the voxel-count weighted average of median uptake values in cortical regions of interest, normalized to cerebellar gray matter. 25 26

The regions analyzed included: prefrontal, anterior cingulate, precuneus, posterior cingulate, parietal, lateral temporal, medial temporal, occipital, sensorimotor, pons, and cerebellum (gray and total matter). A cutoff point of 1.42 for the composite SUVr, using the cerebellar cortex as reference region, was considered as the positivity standard. 23 25 26 The composite SUVr was then used to assess possible correlations with performances obtained on neuropsychological tests.

### FDG-PET and PIB-PET processing and quantitative group analysis

The T1-weighted sequence MRI images were preprocessed with manual removal of extracerebral structures using the MRIcron software (NeuroDebian). The FDG- and PIB-PET images were then coregistered with their respective T1-weighted sequence MRI images using the PMOD software (PMOD Technologies LLC), version 3.4002.

Then, FDG- and PIB-PET images were processed using the Statistical Parametric Mapping 8 (SPM8) software (University College London). Processing steps for PET and MRI data were validated in previous studies. 23 27 The GMV was measured with volumetric T1-weighted sequence MRI, processed by the voxel-based morphometry method (VBM) in SPM8.

The VBM comparisons of PET uptake were then performed using independent samples Student's t-test between two groups. Statistical parametric maps were generated with the SPM8 threshold at the voxel level with *p* (p _unc_ ) = 0.001, uncorrected for multiple comparisons, with a minimum extension of 10 voxels in the cluster. Statistical results were considered valid when they survived correction for multiple comparisons with the family-wise error (p _FWE_ ) and false discovery rate (p _FDR_ ) methods, with the *p* -value set at 0.05. Statistical parametric brain maps comparing the groups for cortical amyloid deposition, rBGM, and GMV were generated, considering schooling as a covariate.

### Statistical analysis

Categorical variables were expressed as absolute and relative frequencies and compared with Pearson's Chi-squared on univariate analysis. Descriptive statistics including mean, SD, median and interquartile range (IQR) values were generated for continuous numerical variables. Variables were tested for normality with histogram graphs and the Kolmogorov-Smirnov test.

For continuous numerical variables with normal distribution, analysis of variance (ANOVA) followed by Tukey's post-hoc test was performed to compare the three clinical groups. Moreover, for variables with non-normal distribution, the Kruskal-Wallis one-way ANOVA followed by Dunn-Bonferroni's post-hoc test were performed. Spearman's correlation coefficient was used to measure the correlation between cognitive scores and SUVr in PET images.

Additionally, multiple linear regression analyses were performed to investigate the independent associations of composite SUVr, age, schooling, and sex with neuropsychological performance. Models were run for RAVLT-DR and Logical Memory II, with all predictors entered simultaneously. No multicollinearity was detected (variance inflation factors < 2.0). Adjusted R ^2^ and standardized β were reported.

The analysis was performed using the IBM SPSS Statistics for Windows (IBM Corp.) software, version 25.0. All tests were performed considering bilateral hypotheses, and two-tailed and *p* -values < 0.05 were considered as statistically significant.

### Standard protocol approvals, registrations, and patient consents

The study was approved by the Ethics and Research Committee of the HCFMUSP under protocol number 62047616.0.0000.0068. Informed Consent was obtained from each participant. The study of human subjects was designed and conducted according to the Declaration of Helsinki.

## RESULTS

### Demographic, clinical, and neuropsychological profile

Demographic and clinical data, and neuropsychological test scores are summarized in Table 1 . The age of SA ranged from 80 to 91 years, with a median of 81 years. The SA group did not differ from the other groups in terms of years of schooling, and it presented a lower frequency of women than the HC60 group. The groups did not differ regarding clinical comorbidities.

**Table 1:** Demographic and clinical information and neuropsychological test scores

Demographic and clinical information	SA (n = 11)	HC80 (n = 23)	HC60 (n = 23)	Post-hoc comparisons ( *p* -value)*		
				SA vs. HC80	SA vs. HC60	HC80 vs. HC60
Age (years): median (IQR)	81.0 (5.0)	83.0 (5.0)	66.0 (3.0)	NS	**< 0.001**	**< 0.001**
Years of schooling: median (IQR)	16.0 (7.0)	16.0 (5.0)	16.0 (5.0)	NS	NS	NS
Female sex: n (%)	5 (45.5)	7 (30.4)	21 (91.3)	**< 0.001**	**< 0.001**	**< 0.001**
Heart disease: n (%)	2 (18.2)	5 (21.7)	2 (8.7)	NS	NS	NS
Hypothyroidism: n (%)	3 (27.3)	3 (13.0)	7 (30.4)	NS	NS	NS
Dyslipidemia: n (%)	2 (18.2)	8 (34.8)	11 (47.8)	NS	NS	NS
Diabetes mellitus: n (%)	2 (18.2)	4 (17.4)	3 (13.0)	NS	NS	NS
SAH: n (%)	5 (45.5)	11 (47.8)	11 (47.8)	NS	NS	NS
GAI: median (IQR)	2.0 (4.0)	2.0 (8.0)	3.0 (5.0)	NS	NS	NS
GDS: median (IQR)	1.0 (2.0)	2.0 (2.0)	1.0 (1.0)	NS	NS	NS
**Neuropsychological test scores**						
RAVLT Delayed Recall:** median (IQR)	12.0 (2.0)	7.0 (3.0)	8.0 (3.0)	**< 0.001**	**0.001**	**0.003**
IQ:** median (IQR)	109.0 (13.0)	107.0 (18.0)	109.0 (15.0)	NS	NS	NS
MMSE: median (IQR)	29.0 (2.0)	29.0 (1.0)	29.0 (1.0)	NS	NS	NS
MoCA: median (IQR)	27.0 (1.5)	24.0 (2.5)	25.0 (2.0)	**0.001**	0.055	**0.282**
BCSB FMT Delayed Recall: median (IQR)	9.0 (2.0)	7.0 (2.0)	9.0 (1.0)	**0.016**	NS	**0.026**
Clock Drawing Test: median (IQR)	9.0 (1.0)	10.0 (1.0)	10.0 (0.0)	NS	NS	NS
Category Verbal Fluency: median (IQR)	19.0 (9.0)	16.0 (6.0)	18.0 (10.0)	NS	NS	NS
Letter Verbal Fluency: median (IQR)	48.0 (19.0)	34.0 (20.0)	38.0 (18.0)	**0.036**	NS	NS
Logical Memory II:** median (IQR)	27.0 (8.0)	18.0 (12.0)	23.0 (7.0)	**0.028**	NS	NS
Rey Figure Copy: median (IQR)	36.0 (0.0)	36.0 (2.0)	36.0 (0.0)	NS	NS	NS
Rey Figure Delayed Recall:** median (IQR)	17.0 (6.5)	10.0 (10.5)	15.0 (10.0)	NS	NS	NS
TMA:** median (IQR)	41.0 (21.0)	52.0 (17.0)	42.0 (12.0)	NS	NS	**0.010**
TMB: median (IQR)	96.0 (69.0)	121.0 (59.0)	80.0 (16.0)	NS	NS	**< 0.001**
Forward Digit Span: median (IQR)	9.0 (2.0)	8.0 (4.0)	8.0 (4.0)	NS	NS	NS
Backward Digit Span: median (IQR)	6.0 (2.0)	5.0 (2.0)	5.0 (2.0)	NS	NS	NS
BNT – 60: median (IQR)	58.0 (7.0)	55.0 (8.0)	58.0 (3.0)	**0.045**	**NS**	**0.039**

Abbreviations: BCSB FMT, Brief Cognitive Screening Battery – Figure Memory Test; BNT, Boston Naming Test; GAI, Geriatric Anxiety Inventory; GDS, Geriatric Depression Scale; HC60, healthy controls aged 60 to 69 years; HC80, age-matched healthy controls; IQ, Intelligence Quotient; IQR, interquartile range; MoCA, Montreal Cognitive Assessment; MMSE, Mini Mental State Examination; NS, not significant; RAVLT, Rey Auditory Verbal Learning Test; SA, SuperAgers; SAH, systemic arterial hypertension; TMA, trail making A; TMB, trail making B; vs., versus.

Notes: *For variables with normal distribution, ANOVA followed by Tukey's post-hoc test was performed. And for variables with non-normal distribution, Kruskal-Wallis one-way analysis of variance followed by Dunn-Bonferroni's post-hoc test was performed; ** Variables with normal distribution.

The SA group showed a higher cognitive performance than the HC80 group, not only in memory tests but also in other cognitive domains tests. Compared to the HC60 group, the SA group showed better results only in the RAVLT-DR.

Interestingly, 6 SA (54.5%) reported SCD. Their memory complaint rate was similar to the HC80 group (52.2%) but smaller than the group to the HC60 group (73.9%). There was no statistically significant difference between SAs with and without SCD regarding the performance on neuropsychological assessment, anxiety, and depression scales.

### Amyloid burden

**Figure 2:**
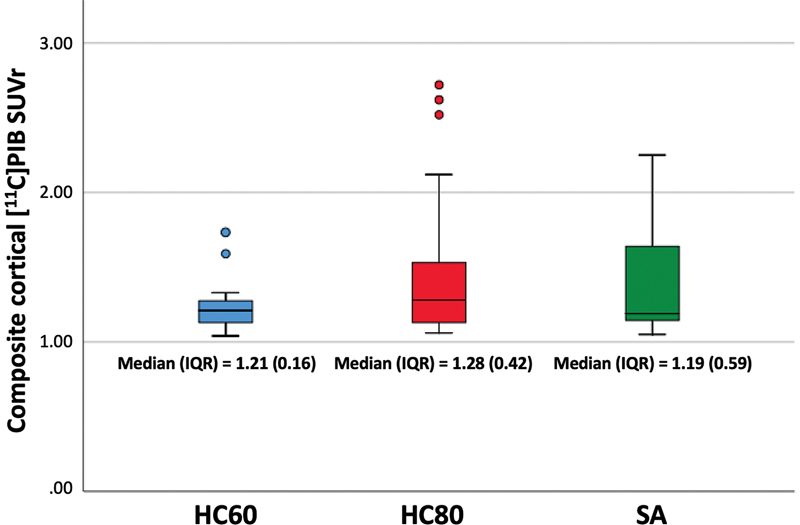
Abbreviations: HC80, age-matched healthy controls; HC60, healthy controls aged 60 to 69 years; IQR, interquartile range; SA, SuperAgers; SUVr, standardized uptake value ratio. Comparison of composite cortical SUVr between SA, HC80, and HC60 groups in PIB-PET ( *p* = 0.392). SUVr > 1.42 (using the cerebellar cortex as the reference region) was considered as positive amyloid status.

In the SA group, 4 participants (36.4%) were PIB-PET positive, without statistically significant differences compared to the HC80 group (n = 9, 40.9%). In contrast, both SA and HC80 groups exhibited a higher percentage of positive PIB-PET than the HC60 group (n = 2, 8.7%). The composite cortical PIB SUVr did not show significant differences between groups ( Figure 2 ). Also, 3 out of 4 SA with PIB-PET also had SCD.

Quantitative group comparisons did not show differences between the SA and HC80 groups, both of which showed a higher uptake than the HC60 group. The SPM8 statistics and areas of increased PIB uptake are detailed in **Supplementary Material Table S1** (available at https://www.arquivosdeneuropsiquiatria.org/wp-content/uploads/2025/10/ANP-2025.0164-Supplementary-Material.docx ).

**Figure 3:**
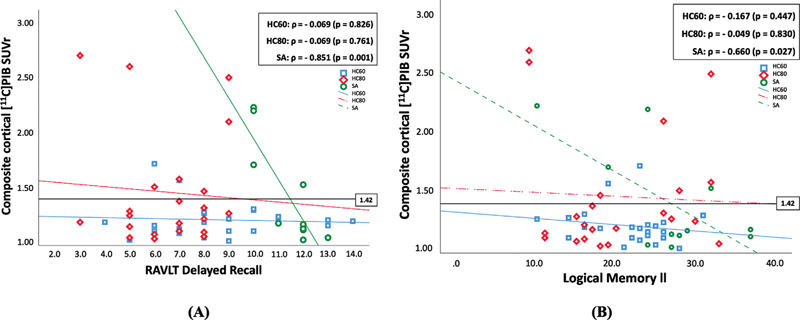
Abbreviations: HC80, age-matched healthy controls; HC60, healthy controls aged 60 to 69 years; SA, SuperAgers. RAVLT, Rey Auditory Verbal Learning Test; SUVr, standardized uptake value ratio. Correlation between RAVLT delayed recall ( **A** ) and Logical Memory delayed recall ( **B** ) and the composite cortical PIB SUVr. An SUVr > 1.42 (using the cerebellar cortex as the reference region) was considered as positive amyloid status.

A negative correlation between memory tests and the composite cortical PIB SUVr value was found in the SA group ( Figure 3 ), but not in the others. Among the SA individuals, those who presented the worst performance in the delayed recall were PIB-PET positive.

Regression analyses indicated that, among SA, higher composite cortical PIB SUVr was significantly associated with lower RAVLT-DR performance (β = −0.666, *p* = 0.011), explaining a substantial proportion of the variance (adjusted R ^2^ = 0.748). No significant association was found in HC80 or HC60. In the full sample, composite cortical PIB SUVr was not related to RAVLT-DR (β = −0.06, *p* = 0.60). Conversely, higher composite SUVr was associated with lower Logical Memory II performance (β = −0.247, *p* = 0.030), though the effect was not present in SA ( Table 2 ).

**Table 2:** Multiple linear regression analyses predicting memory performance (RAVLT-DR and Logical Memory II) from composite SUVr, age, schooling, and sex across groups

Group	Dependent Variable	Predictor	B	SE	β	*p* -value	Adj. R ^2^
**Total (n = 57)**	RAVLT-DR	Composite SUVr	−0.360	0.683	−0.060	0.600	0.001
		Age	−0.007	0.042	−0.023	0.861	
		Schooling	0.099	0.069	0.168	0.154	
		Sex	0.871	0.719	0.162	0.230	
	Logical Memory II	Composite SUVr	−3.953	1.787	−0.247	**0.030**	0.040
		Age	0.032	0.109	0.038	0.767	
		Schooling	0.214	0.180	0.136	0.238	
		Sex	−1.562	1.882	−0.109	0.409	
**SA (n = 11)**	RAVLT-DR	Composite SUVr	−1.534	0.423	−0.666	**0.011**	0.748
		Age	0.013	0.054	0.045	0.816	
		Schooling	0.001	0.059	0.002	0.991	
		Sex	0.795	0.379	0.401	0.081	
	Logical Memory II	Composite SUVr	−11.485	5.847	−0.666	0.097	0.143
		Age	−0.474	0.751	−0.216	0.551	
		Schooling	0.418	0.809	0.186	0.624	
		Sex	2.170	5.228	0.146	0.692	
**HC60 (n = 23)**	RAVLT-DR	Composite SUVr	−0.664	3.355	−0.041	0.845	0.116
		Age	−0.533	0.241	−0.484	**0.040**	
		Schooling	−0.009	0.149	−0.014	0.953	
		Sex	1.748	1.936	0.193	0.378	
	Logical Memory II	Composite SUVr	−4.357	7.420	−0.138	0.564	−00.139
		Age	0.071	0.533	0.033	0.896	
		Schooling	0.261	0.329	0.204	0.438	
		Sex	−0.448	4.281	−0.025	0.918	
**HC80 (n = 23)**	RAVLT-DR	Composite SUVr	−0.068	0.836	−0.020	0.936	−00.155
		Age	0.076	0.108	0.171	0.493	
		Schooling	−0.032	0.116	−0.087	0.786	
		Sex	−0.750	1.169	−0.199	0.530	
	Logical Memory II	Composite SUVr	−0.413	3.476	−0.028	0.907	−00.075
		Age	0.339	0.451	0.177	0.463	
		Schooling	0.211	0.482	0.132	0.668	
		Sex	−3.359	4.860	−0.207	0.499	

Abbreviations: Adj. R ^2^ , adjusted coefficient of determination; B, unstandardized regression coefficient; HC60, healthy controls aged 60 to 69 years; HC80, age-matched healthy controls; RAVLT-DR, Rey Auditory Verbal Learning Test – Delayed Recall; SA, SuperAgers; SE, standard error; SUVr, standardized uptake value ratio; β, standardized regression coefficient. Sex refers to biological sex, coded as 1 = male and 2 = female in the original dataset.

### Regional brain glucose metabolism (rBGM)

In the quantitative group analysis, the SA group had increased rBGM at the left caudate and the left presubgenual anterior cingulate gyrus and did not show areas of reduced rBGM compared to the HC80 group. Conversely, the SA group showed several areas with increased rBGM compared to the HC60 group. Figure 4 illustrates the differences in rBGM and **Supplementary Material Tables S2–S4** show the SPM8 statistics.

**Supplementary Material Tables S2–S7Figure 4:**
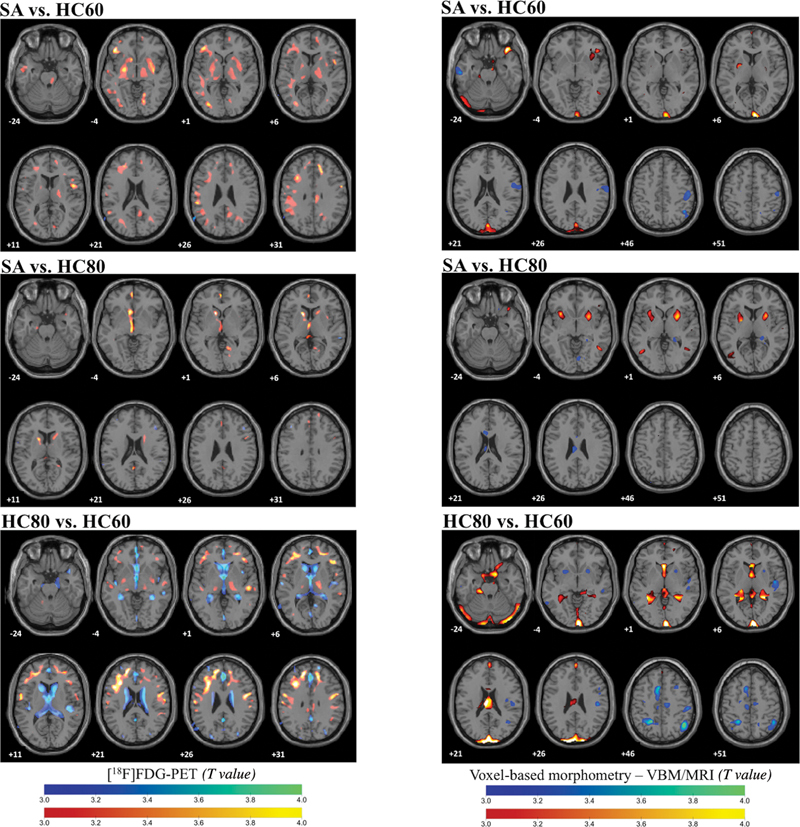
Abbreviations: GMV, gray matter volume; HC80, age-matched healthy controls; HC60, healthy controls aged 60 to 69 years; MRI, magnetic resonance imaging; rBGM, regional brain glucose metabolism; SA, SuperAgers; SPM8, Statistical Parametric Mapping 8. Note: Smaller clusters identified in the lateral ventricles and occipital cortex likely reflect partial volume effects or statistical noise. It is important to emphasize that not all presented clusters survived correction for multiple comparisons (cluster-level family-wise error correction, p _FWE_ < 0.05, as described in the Methods section). Only clusters that met this threshold were interpreted, and full details, including nonsignificant clusters, are presented in . ( **Left** ) Quantitative comparison of rBGM using the SPM8 software between the SA, HC80, and HC60 groups. Areas in red/yellow indicate an increased rBGM and in blue a decreased rBGM. ( **Right** ) Quantitative comparison of GMV measured by volumetric MRI and using the SPM8 software between SA, HC80 and HC60 groups. Areas in red/yellow indicate an increased GMV and in blue a decreased GMV.

**Figure 5:**
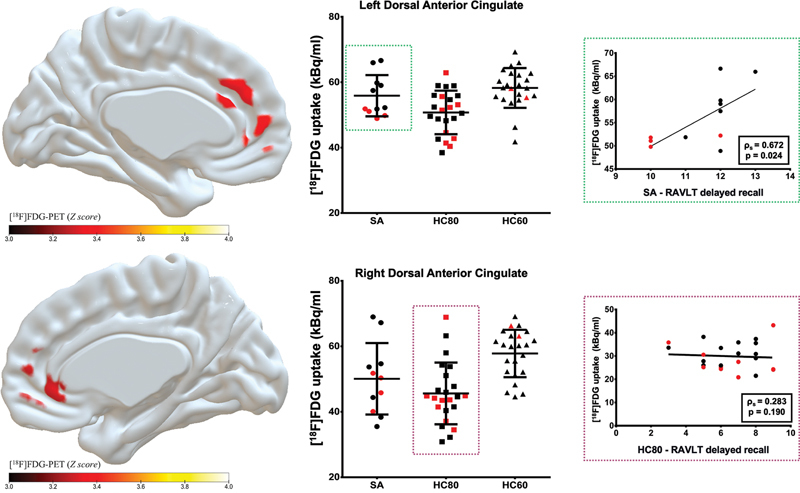
Abbreviations: HC80, age-matched healthy controls; HC60, healthy controls aged 60 to 69 years; IQR, interquartile range; PET, positron-emission tomography; RAVLT, Rey Auditory Verbal Learning Test; rBGM, regional brain glucose metabolism; SA, SuperAgers. ( **A,B** ) Scatter Plot graphical representation showing the dispersion of the ^18^ F-FDG uptake in the clusters of rBGM for each participant (mean radioactive counts), obtained in the group analysis. The dots highlighted in red represent the individual classified as positive amyloid PET. ( **C** ) Graph showing a positive correlation between rBGM and RAVLT delayed recall in a cluster located in the left anterior cingulate.

Regarding the clusters located at the anterior cingulate region with rBGM differences between groups, the SA subjects had an intermediate rBGM average compared to the HC80 and HC60 groups ( Figure 5 ). It is worthy to note that SA participants with positive amyloid PET had lower rBGM at the dorsal anterior cingulate. Additionally, for the SA group, the cluster located in the left anterior cingulate showed a positive correlation between rBGM and RAVLT-DR (r = 0.672, *p* = 0.024).

### Gray matter volume (GMV) and assessment of white matter hyperintensities (WMH)

There was no statistical difference between groups in Fazekas scale. There were 4 SA individuals (36.4%) rated as Fazekas 2, 7 (63.6%) as Fazekas 1, and none was classified as Fazekas 3. There was no association between the WMH and the presence of memory complaints within the SA group.

In the quantitative VBM analysis, the SA group showed increased GMV in the right putamen compared to the HC80 group but no increase compared to the HC60 group ( Figure 4 ). More details on SPM8 statistics can be found in **Supplementary Material Tables S5–S7** .

## DISCUSSION

The present study evaluated 11 SA individuals to search for a neurobiological marker in structural and molecular neuroimaging that could differentiate them from age-matched adults and younger elderly. The main findings were that SA exhibited a similar amyloid burden to that of age-matched older adults, but amyloid deposition negatively impacted memory and rBGM only in SA. In contrast, SA showed areas of increased metabolism in the anterior cingulate gyrus and caudate, as well as increased GMV in the putamen.

There is no consensus about the age in which individuals could be classified as SA, varying between from 60 to over 80 years. 2 3 8 28 29 30 31 32 33 This variability in operational definition leads to heterogeneous results and reflects the need for harmonization. 34 In our study, we used the Northwestern SuperAging research program's criteria, 6 7 which considered those aged 80 years and over as SA. We currently understand SA as an expression of cognitive resilience to physiological and pathological neurobiological changes associated with brain aging that should be investigated among octa- and nonagenarians. 35

Intriguingly, half of the SA had memory complaints, indicating that some of them had SCD. Although this finding did not relate to amyloid burden on PIB-PET, the high rate such complaints in both groups aged ≥ 80 years suggests that SA, like their age-matched peers without exceptional memory, also experience a subjective perception of age-related cognitive decline.

Notably, SA had the same amyloid burden when compared to the HC80 group. The proportion of positive amyloid PET in these 2 groups is congruent with previous studies 28 in populations without dementia aged ≥ 80 years. The relationship between amyloid pathology and exceptional memory performance in SA is controversial in the literature. 36 Some studies showed results similar to ours, 8 10 while in others, SA had a lower amyloid burden, as well as lower tau deposition, as measured by PET. 9 Borelli et al. found that 30% of SA had positive amyloid PET at a frequency similar to age-matched controls. 10 In another cohort of SA and cognitively normal older adults, blood-based biomarkers (Aβ _42_ /Aβ _40_ ratio, tau-181, and tau-181/Aβ _42_ ratio) were used and the two groups showed no differences. 37

In the present sample, although all SA had an exceptional memory, those with positive amyloid PET performed worse in memory tests, suggesting that the amyloid burden negatively influences SA individuals' memory. Nevertheless, the absence of an association between SCD and amyloid PET results in our sample indicated that amyloid pathology was not a single factor that influences the presence or absence of subjective memory complaints.

Dang et al. also showed that those SA subjects with positive amyloid PET had the same rate of cognitive decline over 8 years of follow-up as the non-SA individuals. 3 Dekhtyar et al. showed that SA with positive amyloid PET evolved with more significant cognitive decline in a 3-year follow-up than “amyloid negative” SA. 31 Harrison et al. found that the composite SUVr in PIB-PET predicted cognitive decline in controls but not in SA. 8 Cortical deposition of amyloid is linked to progressive cognitive decline in cognitively normal older individuals, which is one of the evidences that support the amyloid-cascade theory of Alzheimer's disease (AD). 38 Our results suggest SA are not immune to this process.

The aforementioned findings, showing that SA exhibit amyloid burdens similar to age-matched older adults, raise three key points for discussion: that SA are more resilient to the neurobiological effects associated with the pathological changes of AD; that amyloid pathology alone is not sufficient to lead to decline; and that SA have some mechanism of resistance to the development of neurodegeneration.

Regression analyses revealed that, in SA, composite SUVr was a strong and independent predictor of RAVLT-DR performance, explaining nearly 75% of the variance, indicating that amyloid deposition exerts a measurable negative effect on episodic memory.

Regarding cerebral metabolism, the comparative analysis of the rBGM showed that SA had several cortical areas with increased cerebral metabolic activity compared to HC60, mainly those connected through the ventral salience and dorsal executive/salience networks.

The salience network, with nodes centered on the anterior cingulate and the anterior insula, plays a crucial role in integrating sensory stimuli, identifying the motivationally relevant ones and directing attention and behavior to these stimuli. 39 40 This function makes it important to select new information that will be relevant for memorization processes. Therefore, it is a network that participates in the episodic memory system, and these findings may be related to exceptional memory performance in SA. Compared to the HC80, the SA group had an increased rBGM in the anterior presubgenual cingulate cortex (ventral salience network) and left caudate (dorsal executive/salience network).

The increased rBGM in caudate is a finding that has not been previously reported in SA studies, which caught our attention. In line with this finding in FDG-PET, we also found an increased GMV in striatal regions. These findings suggest that striatum is also related to networks involved with episodic memory, such as the hippocampal-centered, 41 42 salience, 39 43 and default mode networks. 42 44 Rieckmann et al. identified that differences in the connectivity of the caudate with the default mode and frontoparietal networks can predict changes in memory over the years. 42 Therefore, our findings suggest that cortico-subcortical loops are also relevant to successful cognitive aging.

Furthermore, in the SA group, the metabolic activity in the anterior cingulate showed a positive correlation with RAVLT-DR. The anterior cingulate cortex is known as a key structure to explain exceptional cognitive performance in SA, 4 6 7 32 connecting the salience and executive central networks. 43 45 Several studies have shown increased rBGM 28 or greater cortical thickness of the anterior cingulate cortex in SA compared to age-matched elderly or even middle-aged adults. 4 6 7 8 32

This study has some limitations. The small sample size, particularly in the SA group, reduces statistical power and generalizability. This precluded the use of more robust modeling approaches, such as general linear models adjusting for covariates. The cross-sectional design precludes conclusions about causality or cognitive trajectories over time. Additionally, the absence of tau pathology biomarkers limits a more complete characterization of AD pathology, primarily because tau pathology is more associated with cognitive decline than amyloid pathology. The exclusion of individuals with low schooling may reduce the representativeness of the sample in a middle-income country with educational heterogeneity, such as Brazil. Finally, the findings regarding increased metabolism and GMV in basal ganglia structures, although intriguing, should be interpreted with caution due to the small sample size and the still limited evidence in the literature, requiring confirmation in future studies.

In conclusion, the present investigated a sample of SA with amyloid burden equivalent to age-matched adults. Amyloid deposition negatively influenced memory performance specifically in the SA group. On the other hand, our SA sample showed increased cerebral metabolism and GMV in the cortical and subcortical components of the salience network. Finally, the most surprising result was the increased metabolism and increased GMV in the basal ganglia, a finding so far unheard of in SA studies.
